# Loss of Tropomodulin4 in the zebrafish mutant *träge* causes cytoplasmic rod formation and muscle weakness reminiscent of nemaline myopathy

**DOI:** 10.1242/dmm.017376

**Published:** 2014-10-02

**Authors:** Joachim Berger, Hakan Tarakci, Silke Berger, Mei Li, Thomas E. Hall, Anders Arner, Peter D. Currie

**Affiliations:** 1 Australian Regenerative Medicine Institute, Monash University, Clayton, VIC 3800, Australia; 2 Department of Physiology and Pharmacology, Karolinska Institute, Stockholm, SE 17177, Sweden; 3 Institute for Molecular Bioscience, University of Queensland, St Lucia, QLD 4072, Australia.

**Keywords:** Myofibrillogenesis, Nemaline myopathy, Neuromuscular disorder, Sarcomere assembly, *tmod*, Tropomodulin

## Abstract

Nemaline myopathy is an inherited muscle disease that is mainly diagnosed by the presence of nemaline rods in muscle biopsies. Of the nine genes associated with the disease, five encode components of striated muscle sarcomeres. In a genetic zebrafish screen, the mutant *träge* (*trg*) was isolated based on its reduction in muscle birefringence, indicating muscle damage. Myofibres in *trg* appeared disorganised and showed inhomogeneous cytoplasmic eosin staining alongside malformed nuclei. Linkage analysis of *trg* combined with sequencing identified a nonsense mutation in *tropomodulin4* (*tmod4*), a regulator of thin filament length and stability. Accordingly, although actin monomers polymerize to form thin filaments in the skeletal muscle of *tmod4^trg^* mutants, thin filaments often appeared to be dispersed throughout myofibres. Organised myofibrils with the typical striation rarely assemble, leading to severe muscle weakness, impaired locomotion and early death. Myofibrils of *tmod4^trg^* mutants often featured thin filaments of various lengths, widened Z-disks, undefined H-zones and electron-dense aggregations of various shapes and sizes. Importantly, Gomori trichrome staining and the lattice pattern of the detected cytoplasmic rods, together with the reactivity of rods with phalloidin and an antibody against actinin, is reminiscent of nemaline rods found in nemaline myopathy, suggesting that misregulation of thin filament length causes cytoplasmic rod formation in *tmod4^trg^* mutants. Although Tropomodulin4 has not been associated with myopathy, the results presented here implicate*TMOD4* as a novel candidate for unresolved nemaline myopathies and suggest that the *tmod4^trg^* mutant will be a valuable tool to study human muscle disorders.

## INTRODUCTION

Congenital myopathies (CMs) are a heterogeneous group of muscle disorders characterized by muscle weakness, hypotonia and delayed motor milestones usually present from birth. In contrast to dystrophies, which typically feature degenerating myofibres and fibrosis, CMs often present with impaired functional myofibres without clear signs of degeneration and regeneration (Bönnemann et al., 2014). Many genes have been identified as provoking congenital myopathy in human; however, the genetic and molecular basis of many muscle diseases still remain unresolved (Kaplan and Hamroun, 2013). Although CMs substantially overlap in their clinical and genetic features, they are categorized into five main types (North et al., 2014): (1) nemaline myopathy (NM) that historically has been defined by the presence of cytoplasmic rods, (2) core myopathy that in addition to nemaline rods features cores of accumulated myofibrillar material, (3) centronuclear myopathy, (4) myosin storage myopathy, and lastly (5) congenital fibre type disproportion.

NM presents with a broad clinical spectrum ranging from fatal forms with neonatal onset to mild non-progressive forms with onset at adulthood. However, all forms of NM are diagnosed by the presence of electron-dense amorphous threads in myofibres, named nemaline rods (Schnell et al., 2000). Nemaline rods show a type I (slow) fibre predominance in mild cases of the disease and a broad distribution throughout the muscle in severe forms of NM (Malfatti et al., 2014). Nemaline rods vary in number, size and shape, are typically in structural continuity with the sarcomere Z-disk and at times appear as thickened Z-lines. Accordingly, these rods show an ultrastructure resembling the lattice pattern of the Z-disk and immunohistochemically stain with antibodies against actinin and other proteins of the Z-disk, suggesting that misregulation of Z-disk assembly or maintenance might be crucial in the disease pathology. In line with this assertion, of the nine genes identified to be associated with NM to date (Gupta et al., 2013), five encode structural components of the sarcomere, including *ACTA1*, *NEB*, *TPM2*, *TPM3* and *TNNT1* (Donner et al., 2002; Johnston et al., 2000; Laing et al., 1995; Nowak et al., 1999; Pelin et al., 1999).

A major component of skeletal muscle sarcomeres is actin thin filaments that have a barbed and pointed end. At the barbed end, thin filaments are capped by CapZ and locked into the sarcomere Z-disk. At the pointed end, thin filament formation is concluded by tropomodulins that cap the filaments and thereby regulate their length and stability (Almenar-Queralt et al., 1999; Fowler et al., 1993; Gokhin and Fowler, 2013). The N-terminus of Tmod1 also binds to tropomyosin, another thin filament interacting partner that enhances the filament capping activity of tropomodulins (Rao et al., 2014; Yamashiro et al., 2012). Loss of Tmod1 function in mice leads to reduced isometric force production by the muscle, and the proportion of fast fibre types is increased at the expense of slow fibres (Gokhin et al., 2010). Interestingly, however, the sarcomeres are structurally preserved as Tmod1 is replaced by Tmod3 and Tmod4 (Gokhin et al., 2010); therefore, Tmod proteins have not been associated with NM to date.

**RESOURCE IMPACT**

**Background**

Skeletal muscle has many vital functions, such as production of locomotion, maintenance of posture, regulation of body temperature, and breathing. Owing to its pivotal role, diseases that involve skeletal muscle have severe symptoms. Congenital myopathies are inherited muscle disorders with a variety of symptoms, including muscle weakness, hypotonia (decreased muscle tone) and delayed motor milestones (child development stages) that are usually present from birth. Unravelling the genetic and molecular basis of inherited diseases is fundamental for the development of therapies. However, for nemaline myopathy – a form of myopathy associated with the presence of thread-like rods, called nemaline bodies, in muscle cells – it is estimated that for approximately 25% of the cases, the genetic basis is still unresolved. To newly identify genes involved in this myopathy, this study used a novel zebrafish muscle mutant named *träge*.

**Results**

In a forward genetic screen, the zebrafish *träge* mutant showed defective musculature. In particular, the swim bladder did not inflate and the impaired swimming behaviour, together with the severe muscle weakness, left mutants unable to hunt for food, leading to starvation at 11–12 days post fertilisation. Gene mapping and subsequent gene analysis identified a loss-of-function allele in the *tropomodulin4* gene (*tmod4*) in this mutant. In skeletal muscle, tropomodulins cap actin thin filaments and thereby regulates their length and stability. Because of the loss of Tmod4 activity, sarcomeres of the contractile apparatus of the mutant appeared disorganised, with altered features such as widened Z-disks and undefined H-bands (two important substructures of the sarcomeric unit). Importantly, cytoplasmic rods were identified in the mutant myofibrils that, in their lattice pattern and protein content, resembled nemaline rods found in individuals suffering from nemaline myopathy.

**Implications and future directions**

Over the last two decades, the zebrafish has received tremendous attention owing to its advantages as a model system and the fact that zebrafish models of human diseases often closely resemble the human pathology. The phenotype of the novel zebrafish mutant *tmod4*^trg^ identifies *tmod4* as a novel candidate gene for unresolved myopathies. In addition, *tmod4*^trg^ mutants can be used to study the role of thin filament capping in sarcomere assembly and in the formation of cytoplasmic rods, which underlie the diagnosis of nemaline myopathy in humans. Importantly, the zebrafish is also unique amongst vertebrate model systems because its efficient husbandry and high level of fecundity enable high-throughput screening of small-molecule-based therapies. Such research could not only lead to novel insights into the molecular basis that underlies the pathology of myopathies, but could also potentially help to develop novel compounds for the treatment of human muscle disorders.

To newly identify genes involved in myopathies, we performed a 1-ethyl-1-nitrosourea (ENU)-based genetic screen in zebrafish, as zebrafish mutants often closely resemble the pathogenesis of human myopathies (Berger and Currie, 2012). Screening for a reduction in birefringence, a marker for muscle damage (Berger et al., 2012), identified the muscle mutant named *träge* (*trg*). Using brightfield microscopy analysis, the *träge* mutant appears similar to siblings, whereas polarised light highlights a severe reduction in birefringence, indicative of muscle damage. Single nucleotide polymorphism (SNP)-based mapping and subsequent sequencing of *trg* identified a nonsense mutation in *tmod4*, which was verified as causing the reduction in birefringence seen in *trg* mutants. Haematoxylin and eosin (H&E)-stained cross sections of *tmod4^trg^* mutants also indicated muscle damage, and a reduced contractile apparatus confirmed in the double transgenic background of *Tg*(*acta1:lifeact-GFP*) and *Tg*(*acta1:CherryCAAX*), marking the contractile apparatus of the myofibre and sarcolemma, respectively (Riedl et al., 2008). Thin filaments in the skeletal muscle of *tmod4^trg^* varied in length, often appeared in crisscross pattern, and were rarely integrated into organised sarcomeres. Accordingly, the musculature of *tmod4^trg^* had a reduced isometric strength, as quantified by mechanical experiments. Importantly, the myofibril featured widened Z-disks, undefined H-bands, and an abundance of cytoplasmic rods, which were also detected on Gomori-stained sections, that are reminiscent of nemaline rods. We therefore suggest that the zebrafish mutant *tmod4^trg^* is a novel model for human myopathies, which can be used to study cytoplasmic rod formation, and that *TMOD4* is novel candidate gene for unresolved human myopathies.

## RESULTS

### Isolation of the novel zebrafish muscle mutant *träge*

**Fig. 1.**
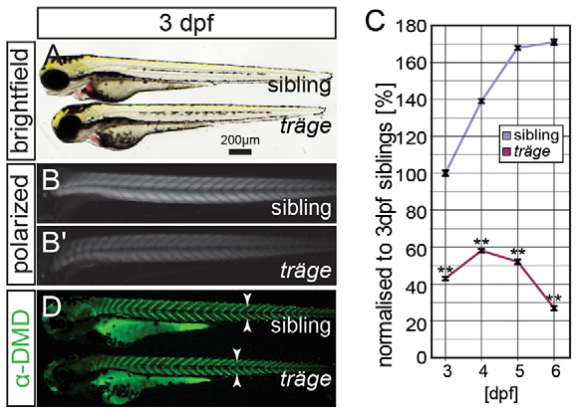
**The mutant *träge* (*trg*) shows a reduction in birefringence, indicating muscle damage.** (A) Under brightfield microscopy, *trg* mutants appear similar to their wild-type siblings. (B) Under polarised light, the muscle of siblings appears brighter than that of the (B′) *trg* mutants due to a reduction in birefringence. (C) Quantification of the birefringence followed by normalization to that of 3-dpf-old siblings reveals that the birefringence of the siblings increases from 3 dpf to 6 dpf, roughly following a sigmoidal curve. By contrast, 3-dpf- to 6-dpf-old *trg* larvae show a highly significant reduction in birefringence when compared with that of 3-dpf siblings (*P*<0.01, *n*=3). (D) Immunohistochemistry with antibodies against dystrophin shows that dystrophin expression at the vertical myosepta (arrowheads) is unaffected in *trg* mutants. Data are means±s.e.m., **P<0.01. Scale bar: 200 μm.

To identify novel genes associated with congenital muscle diseases, a forward genetic screen was performed aiming to isolate zebrafish mutants with defects in the musculature. The chemical ENU was used to induce random mutations in males, which were outcrossed over two generations to establish 126 F2 families (Berger et al., 2011). The progeny of F2 incrosses were screened under polarised light at 3 days post fertilization (dpf). Polarised light shows the birefringence of muscle, a light effect provoked by the pseudo-crystalline array of the sarcomeres of the contractile apparatus; therefore, a reduction in birefringence indicates a broad range of myofibre defects and correlates with the level of muscle damage (Berger et al., 2012). One mutant was isolated that appeared similar to siblings under brightfield conditions. The total body length of mutant larvae was only slightly reduced to 96.0±0.6% compared with their siblings, and the swimming bladder did not inflate (*P*<0.01, *n*=7). In addition, mutants were sensitive to touch and showed an impaired motility with compromised forward thrust (supplementary material Movies 1, 2), which led to the designation *träge* (*trg*) (German for slow). Under polarised light, *trg* mutants displayed a marked reduction in birefringence (Fig. 1A–B′). At 3 dpf, there was a highly significant reduction in the birefringence of *trg* mutants to 45±2% of that of siblings, which persisted through to 6 dpf (*P*<0.01, *n*=3) (Fig. 1C). The birefringence of *trg* homozygotes normalised to that of siblings at 3 dpf were 58±2% at 4 dpf, 52±2% at 5 dpf, and 27±2% at 6 dpf (*P*<0.01, *n*=3). Importantly, the birefringence of *trg* homozygotes remained uniform rather than scattered, as has been seen in dystrophic mutants, in which the scattered distribution of detaching and degenerating fibres leads to a patchy pattern of birefringence (Fig. 1B′) (Berger et al., 2012). As birefringence is provoked by the myofibril, this indicates that the reduction in birefringence of *trg* mutants comes from a defect in the myofibril, rather than a stochastic loss of entire myofibres due to degeneration. Similarly, dystrophin expression at the vertical myosepta of *trg* mutants, analysed by immunohistochemistry using antibodies against dystrophin, matched that of siblings, revealing that myofibre differentiation is unaffected (Fig. 1D). Nonetheless, although siblings were viable and fertile, *trg* mutants died by 11 to 13 dpf. It is probable that an impaired swimming ability leaves *trg* mutants unable to hunt effectively for food, as starved siblings died at the same age.

Taken together, *trg* mutants carry a recessive lethal mutation that causes severe muscle weakness and a uniform reduction in birefringence, indicating deficiencies in the contractile apparatus of the muscle.

### *Träge* mutants harbour a nonsense mutation in *tropomodulin4*

**Fig. 2.**
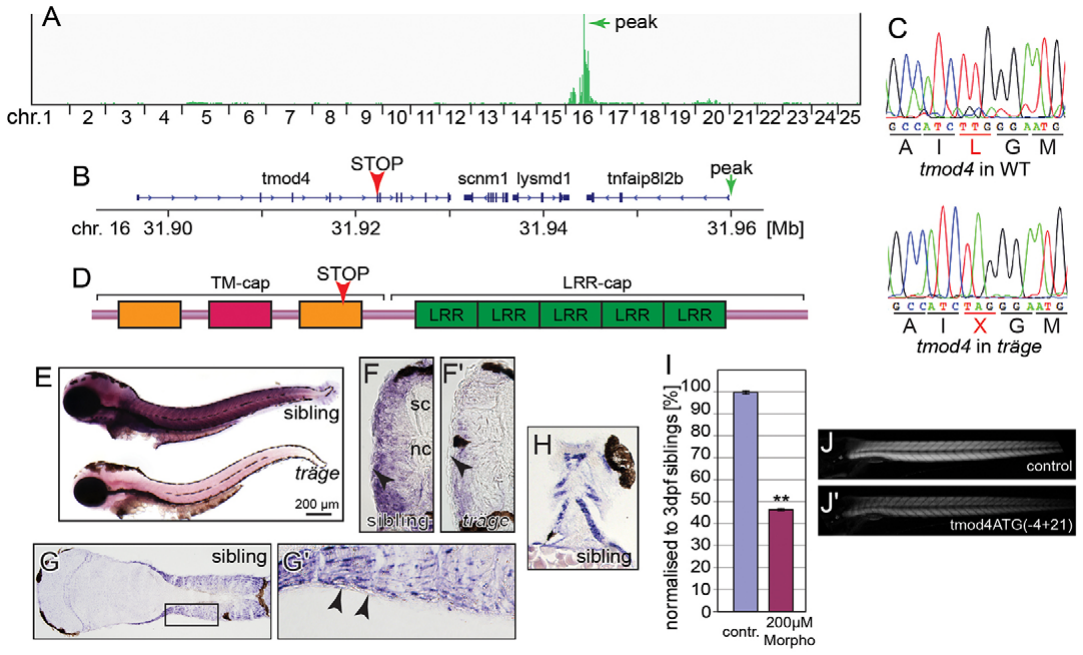
***Träge* carries a *tmod4* loss-of-function allele.** (A) Linkage analysis of *trg* mutants through SNPtrack resulted in a peak at 31.96 Mb on chromosome (chr.) 16 (marked by green arrow). (B) Within the locus lies *tmod4* that carries a nonsense mutation, indicated by a red arrowhead. (C) The triplet TTG that encodes a leucine residue in wild-type *tmod4* is mutated to the stop codon TAG in *tmod4^trg^* mutants (L132X). (D) Schematic of Tmod4 shows the tropomyosin and actin-capping domain (TM-cap) that comprises two tropomyosin-binding helices (orange) and one actin-binding helix (red) together with the actin-binding domain (LRR-cap) with its five leucine-rich repeats. The arrowhead marks the location of the L132X mutation. (E) Whole-mount *in situ* hybridization at 3 dpf shows an abundance of *tmod4* transcript in trunk muscle that is strikingly reduced in *tmod4^trg^*, suggesting nonsense-mediated decay. (F,F′) The weaker *in situ* signal in *tmod4^trg^* is also noted on cross sections. Interestingly, *tmod4* transcript is absent from the superficial slow muscle fibres (arrowheads), spinal chord (sc) and notochord (nc). Longitudinal sections show muscle-specific *tmod4* transcript in the (G,G′) trunk and (H) head musculature. G′ shows an enlarged image of the region of interest in G. (I–J′) Administration of 200 μM of tmod4ATG(−4+21) into wild-type embryos phenocopied *tmod4^trg^* mutants by inducing a highly significant reduction in birefringence after normalization to control-injected embryos (*P*<0.01, *n*=3). (I) Quantification results, data are means±s.e.m., **P<0.01. (J,J′) example images. contr., control; WT, wild type.

In order to identify the phenotype-causing mutation in *trg*, mutants were subjected to positional cloning based on SNPs. Genomic DNA was isolated from pools of 25 phenotypic *trg* mutants and 25 siblings, the DNA of each pool was then sequenced using next-generation sequencing. SNP variants in the generated sequencing reads were detected by using the SNPtrack software, and identified regions of homozygosity were integrated in a linkage map that was visualized by using the software Integrative Genomics Viewer (IGV) (Fig. 2A) (Leshchiner et al., 2012; Robinson et al., 2011). Linkage analysis resulted in a main peak located on chromosome 16 at 31.96 Mb (Fig. 2B). Further sequencing of the genes in the identified locus revealed a nonsense mutation in exon 5 of *tropomodulin4* (*tmod4*) in *trg* mutants. In this *tmod4^trg^* allele, the triplet TTG encoding a leucine residue in one of the tropomyosin-binding domains was mutated to the stop codon TAG (L132X) (Fig. 2C). Tropomodulin4 is a protein of 343 amino acids that caps thin filaments in skeletal muscle (Almenar-Queralt et al., 1999; Gokhin et al., 2010). We therefore believe that the discovered premature stop codon L132X is likely to provoke the muscle phenotype of *tmod4^trg^*. In order to substantiate that the nonsense mutation in *tmod4* causes the *tmod4^trg^* phenotype, PCR-based genotyping of 96 *tmod4^trg^* mutants and 96 siblings was performed to determine whether the mutation in *tmod4* segregates with the *tmod4^trg^* phenotype. This genotyping revealed that all phenotyped *tmod4^trg^* mutants were homozygous carriers of the identified nonsense mutation, and siblings were either heterozygous or wild-type *tmod4* carriers, confirming that *tmod4^trg^* mutants harbour a nonsense mutation in *tmod4*. For subsequent experiments, *tmod4^trg^* mutants were genotyped by using the same protocol.

Next, *in situ* hybridization was performed to analyse *tmod4* expression. A strong signal was detected in siblings at 3 dpf that appeared to be markedly reduced in *tmod4^trg^* mutants, which we believe is due to nonsense-mediated decay (Fig. 2E). Correlating with the muscle phenotype of *tmod4^trg^*, abundant amounts of *tmod4* transcript was found in the trunk and head musculature (Fig. 2F,H). Interestingly, slow fibres that form a superficial layer on the lateral side of somites in zebrafish were devoid of signal (Fig. 2F,G). Consistent with nonsense-mediated decay, neither full nor truncated Tmod4 protein was detected by using western blot analysis of *tmod4^trg^* mutants (supplementary material Fig. S1), indicating that *tmod4^trg^* is a loss-of-function mutant.

To phenocopy the *tmod4^trg^* phenotype, knockdown experiments using morpholino-antisense-oligonucleotides targeting the translation initiation codon of the *tmod4* transcript were performed. Injection of the morpholino tmod4ATG(−4+21) at a concentration of 200 μM into wild-type embryos induced a highly significant reduction in birefringence to 46±1% when normalised to that of control-injected siblings (Fig. 2I–J′) (*P*<0.01, *n*=3), a phenotype reminiscent of *tmod4^trg^* mutants.

In conclusion, the phenotype of *tmod4^trg^* results from a null allele of *tmod4*, a known regulator of thin filament dynamics in skeletal muscle.

### *Tmod4^trg^* mutants show reduced amounts of myofibril

**Fig. 3.**
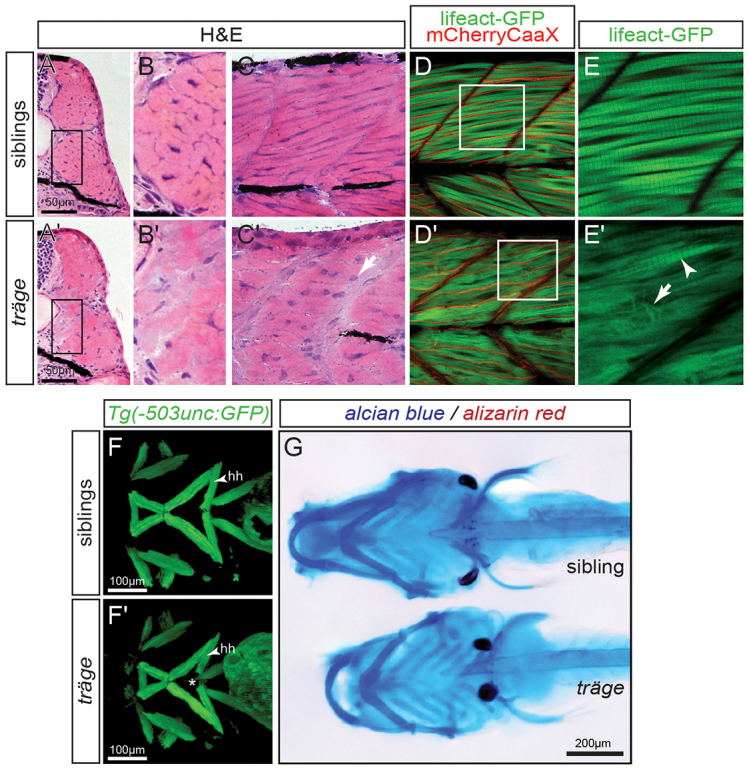
**Muscle phenotype of *tmod4^trg^*.** (A) In comparison to their siblings, (A′) myofibres in H&E-stained cross sections of *tmod4^trg^* larvae show less defined cell shapes and inhomogeneous eosin staining at 3 dpf. (B,B′) Magnified views of the respective boxes indicated in A and A′. (C,C′) Accordingly, myofibres on sagittal sections of *tmod4^trg^* mutants appear unstructured, and blue haematoxylin-stained nuclei (arrow) are rounder in shape compared with those of siblings. (D) In a sagittal view of 3-dpf-old siblings, the myofibrils show typical striation, when marked by using the transgenic line *Tg*(*acta1:lifeact-GFP*). In addition, the transgenic background *Tg*(*acta1:mCherryCAAX*) labels the sarcolemma in red, showing that myofibrils occupy most of the myofibres. (D′) In matching *tmod4^trg^* mutants, the myofibril striation is rarely seen (arrowhead in E′), instead myofibres are filled with misorientated thin filaments (arrow in E′). (E,E′) Magnifications of respective boxes in D and D′. (F,F′) GFP highlights the cephalic musculature in the transgenic background of *Tg*(*−503unc:GFP*), here shown as ventral views of *z*-stack projections. (F) In contrast to their siblings, (F′) the two hyohyoideus (hh) muscles in *tmod4^trg^* mutants leave a gap between each other (asterisk). (G) Focus stacks of larvae that were stained with Alcian blue and Alizarin red depict cartilage malformations in *tmod4^trg^* at 6 dpf.

As the detected reduction in birefringence in *tmod4^trg^* mutants is indicative of a defective contractile apparatus, the muscle of *tmod4^trg^* homozygotes was histologically assessed. To characterise the muscle histology of *tmod4^trg^* mutants, cross and sagittal sections of larvae at 3 dpf were stained with H&E. In comparison with siblings, *tmod4^trg^* homozygotes showed disorganised myofibres with less defined cell shapes, and cytoplasmic eosin staining appeared inhomogeneous (Fig. 3A–C′). In addition, the haematoxylin-stained nuclei of *tmod4^trg^* mutants appeared rounder in shape compared with those of siblings. Interestingly, the muscle of *tmod4^trg^* mutants was only slightly reduced in size. The skeletal muscle cross-sectional area (CSA) of 3-dpf-old siblings was 0.0328±0.0005 mm^2^, and that of *tmod4^trg^* mutants was 0.0309±0.0004 mm^2^ (equalling a reduction to 94±1%, *P*<0.01, *n*=4).

In order to survey myofibril organisation in live zebrafish embryos in more detail, two transgenic lines were generated: *Tg*(*acta1:lifeact-GFP*), which marks thin filaments by using the Lifeact green fluorescent protein (Lifeact-GFP) fusion protein that binds to thin filaments through the Lifeact tag (Riedl et al., 2008); and *Tg*(*acta1:mCherryCAAX*), which highlights the sarcolemma by integration of mCherry-CAAX enforced by the CAAX tag. As already indicated by the reduction in birefringence, bundles of organised myofibrils with their typical striation were rarely detected in double-transgenic *tmod4^trg^* mutants (Fig. 3D–E′). Instead, thin filaments marked by Lifeact-GFP were not organised into sarcomeres and appeared to be misoriented (Fig. 3E′). In addition, labelling of the sarcolemma revealed that unorganised thin filaments were abundant and dispersed throughout myofibres (Fig. 3D′).

To analyse whether the head musculature of *tmod4^trg^* was affected, *tmod4^trg^* mutants were crossed into the transgenic background of *Tg*(*−503unc:GFP*), which expresses GFP throughout the zebrafish musculature (Berger and Currie, 2013). Although all head muscles appeared anatomically normal in *tmod4^trg^* homozygotes, the GFP signal illustrated a gap between the two contralateral hyohyoideus muscles, as depicted in *z*-stack projections (Fig. 3F,F′). Also, cartilage malformations were exposed by Alcian blue staining in *tmod4^trg^* mutants at 6 dpf (Fig. 3G). As altered muscle strength is known to cause cartilage abnormalities, the detected cartilage malformations were probably caused by muscle weakness.

Taken together, these results show that thin filaments in *tmod4^trg^* mutants are misoriented and are rarely assembled in striated myofibrils, causing muscle weakness.

### Cytoplasmic rods detected in *tmod4^trg^* resemble those in nemaline myopathy

**Fig. 4.**
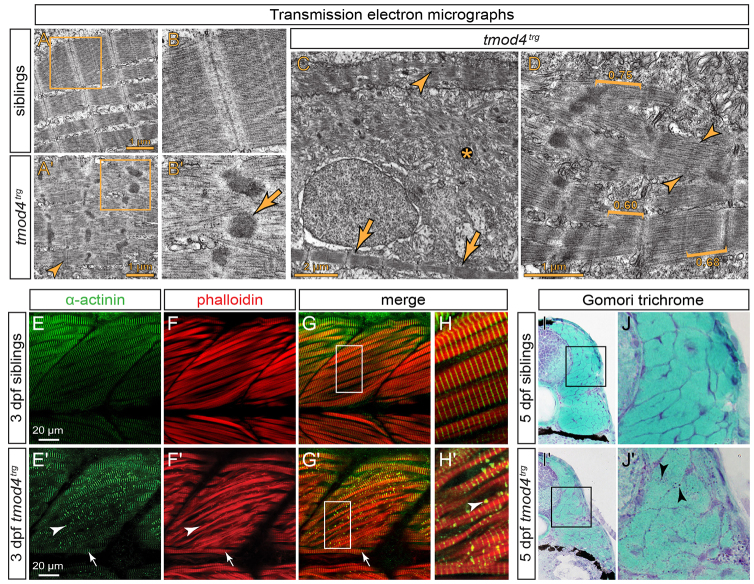
**Cytoplasmic rod formation in *tmod4^trg^*.** (A) Transmission electron micrographs of skeletal muscle from siblings at 3 dpf display the typical myofibril striation and well-aligned sarcomeres. (A′) In contrast, the myofibrils of *tmod4^trg^* mutants appear non-uniform, and filaments are misoriented. Sarcomere H-zones are less defined (arrowhead) and Z-disks are widened with electron-dense inclusions of various sizes and shapes. (B,B′) Magnified views of the respective boxes in A and A′ reveal the lattice pattern of the cytoplasmic rods (arrow) that is typical for nemaline rods of individuals with NM. (C) In addition to abnormal sarcomeres (arrowhead), organised sarcomeres (arrows) rarely form in *tmod4^trg^*, and filaments are often scattered throughout myofibres (asterisk). (D) Brackets mark the various lengths of thin filaments of *tmod4^trg^* from short (0.60 μm) to long (0.75 μm). In organised myofibrils, thin filaments are of lengths comparable to those of siblings (0.68 μm). Indistinct H-zones are marked by arrowheads. (E–H) At 3 dpf, labelling of F-actin with phalloidin (red) and actinin using an antibody (green) shows the typical myofibril striation in siblings. (G,H) Merged images, H shows magnification of the boxed area indicated in G. (E′-H′) In *tmod4^trg^* mutants, actinin and actin colocalize in cytoplasmic aggregations. In contrast to the internal fast myofibres that show abundant cytoplasmic rods (arrowheads), the superficial slow muscle fibres, which in zebrafish form a single layer on the lateral outline of the somites, do not display cytoplasmic rods (arrows). (G′,H′) Merged images, H′ shows magnification of the boxed area indicated in G′. (I,I′) On cross sections at 5 dpf, Gomori trichrome staining indicates the presence of cytoplasmic rods in *tmod4^trg^* that are reminiscent of nemaline rods. (J,J′) Magnifications of boxes indicated in I and I′, respectively. Arrowheads mark nemaline-like cytoplasmic rods.

As tropomodulins play a major role in thin filament length and dynamics (Gokhin and Fowler, 2013; Littlefield et al., 2001), thin filament organisation was analysed in greater detail. In line with the analysis in animals carrying transgenic *Tg*(*acta1:lifeact-GFP*), transmission electron micrographs confirmed that monomeric actin polymerises to form thin filaments in *tmod4^trg^* mutants (Fig. 4A,A′). However, filaments were often scattered throughout the myoplasm and rarely assembled into organised myofibrils (Fig. 4C). Instead, most myofibrils appeared disorganised in *tmod4^trg^* mutants with undefined H-bands and widened Z-disks (Fig. 4D). Electron-dense aggregations of various sizes and shapes were often detected with a clearly documented lattice pattern, all features of nemaline rods documented in muscle biopsies of NM patients (Fig. 4A,A′). The rare organised myofibrils evident in *tmod4^trg^* mutants contained sarcomeres and thin filaments of similar length compared with those of siblings (*tmod4^trg^*, 1.75±0.03 μm; siblings, 0.68±0.01 μm). However, thin filaments of different lengths were measured in misorganised myofibrils. Assuming that, in misorganised myofibrils, thin filaments stretch from Z-disk to the rim of the H-zone, thin filaments of various lengths, including 0.6 μm (short) and 0.75 μm (long), were measured (Fig. 4D).

In order to compare the cytoplasmic rods of *tmod4^trg^* homozygotes to nemaline rods, the composition of the cytoplasmic aggregates was analysed by using immunohistochemistry. Phalloidin staining of F-actin, and antibodies against actinin detected the typical myofibril striation in siblings. In *tmod4^trg^* mutants, however, immunohistochemistry revealed abundant cytoplasmic rods containing actin and actinin, a diagnostic feature of nemaline rods (Fig. 4E–H′). Interestingly, cytoplasmic rods were only detected in fast muscle and were excluded from the superficial slow muscle that, in zebrafish, form a single layer on the outside of somites (Fig. 4E′-G′). This is in line with the expression pattern of *tmod4* that is confined to the fast muscle, as documented by using *in situ* hybridization (Fig. 2). In addition, Gomori trichrome staining of 5-dpf-old sections revealed numerous cytoplasmic rods, similar to nemaline rods (Fig. 4I–J′).

In summary, the sarcomeres in *tmod4^trg^* mutants feature widened Z-disks and abundant cytoplasmic rods with characteristics reminiscent of nemaline rods.

### *Tmod4^trg^* muscles have impaired force generation and altered length-force behavior

**Fig. 5.**
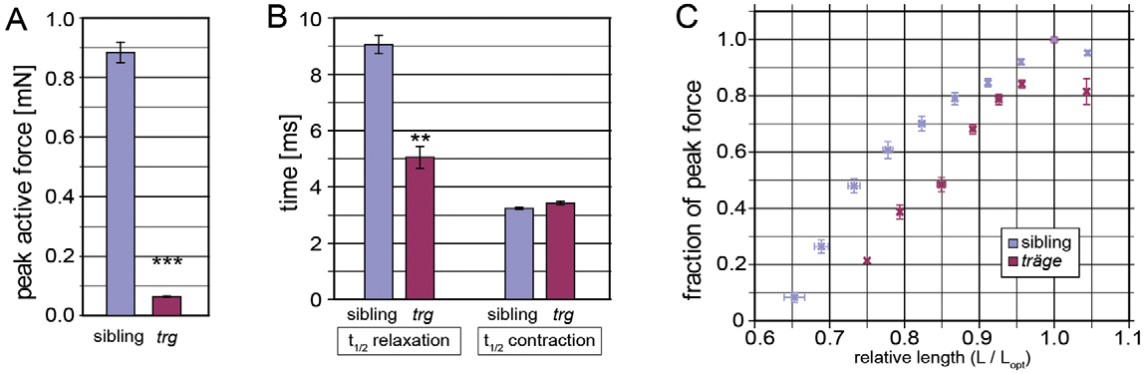
**The *tmod4^trg^* skeletal muscle has impaired force generation.** (A) Peak active force at optimal length in *tmod4^trg^* is significantly lower than that of siblings (*n*=6). (B) During single-twitch contraction, the half-time (t_1/2_) of contraction is similar in *tmod4^trg^* and siblings, however, the relaxation time is significantly prolonged in *tmod4^trg^* (*n*=6). (C) Length–active force curves show steeper ascending limb in *tmod4^trg^* (red) than in siblings (blue) (*n*=5). Force is normalised to the maximal active force at optimal length (L_opt_), and length is normalised to L_opt_. Data are means±s.e.m., **P<0.01, ***P<0.001.

To further investigate the functional deficits in the skeletal muscle of *tmod4^trg^* homozygotes and to quantify their muscle weakness, mechanical experiments were performed using a specialized force transducer (Li et al., 2013). Isometric force and length–active force relations were determined in the trunk muscle of *tmod4^trg^* mutants and siblings at 5 dpf. The muscles were stimulated to give single twitch contractions at different lengths. Consistent with reduced amounts of myofibril, the *tmod4^trg^* trunk muscles generated an isometric strength of 0.064±0.004 mN that was significantly less compared to that of their siblings, which generated 0.884±0.069 mN (*P*<0.001, *n*=6) (Fig. 5A). To determine whether the lower active force emanated from smaller muscle size, the CSA was measured at 5 dpf. Consistent with measurements at 3 dpf, the muscle of *tmod4^trg^* mutants was only very slightly reduced in size; the CSA of sibling muscle was 0.0324±0.0007 mm^2^ and that of *tmod4^trg^* mutants was 0.0315±0.0004 mm^2^ (equalling a reduction to 97±1%, *P*<0.01, *n*=4). Thus, the markedly lower active force evident in *tmod4^trg^* mutants cannot be explained by a decrease in CSA (Fig. 5A).

To further analyse the force transients of the single-twitch, the half-time of contraction and relaxation was measured in both genotypes. The rate of relaxation in *tmod4^trg^* muscles was significantly slower (5.04±0.78 ms) compared to that of their siblings (9.05±0.64 ms) (*P*<0.001, *n*=6), the rate of contraction remained unchanged (3.24±0.07 ms in siblings; 3.43±0.12 ms in *tmod4^trg^*; *n*=6) (Fig. 5B). Plotting normalised isometric force against relative length to analyse the length-force relationships resulted in a bell-shaped curve in both genotypes. However, compared with siblings, the ascending limb of the length-force curve was steeper in *tmod4^trg^* mutants (Fig. 5C). As described in Materials and Methods, the descending limb was not further characterized.

In conclusion, *tmod4^trg^* mutants have severely reduced force generation with altered responses at short muscle length and impaired relaxation, consistent with the drastically reduced amount of myofibril and impaired swimming behaviour evident in these mutants.

## DISCUSSION

Unravelling the genetic and molecular basis of inherited diseases is fundamental for the development of therapies. To date, 360 genes have been associated with inherited monogenic neuromuscular disorders in human, however, many more remain unresolved with at least 92 diseases having been mapped to novel loci (Kaplan and Hamroun, 2013). Specifically for NM, it has been suggested that approximately 25% of cases are genetically unresolved, a relatively coarse estimation due to the overlap of clinical symptoms between different myopathies (North and Ryan, 1993). In an attempt to newly identify candidate genes for congenital skeletal muscle diseases, we performed a genetic screen to isolate zebrafish mutants with musculature defects. In order to rapidly and efficiently screen larvae for muscle damage, a birefringence assay was utilized, as this readily deployable effect of light indicates myofibre atrophy, detachment and thinning (Berger et al., 2012). The screen identified a muscle mutant through a highly significant reduction in birefringence, and SNP-based linkage analysis led to the identification of a nonsense mutation in *tmod4*. PCR-based genotyping, muscle-specific expression of *tmod4*, as well as morpholino-based phenocopy experiments all emphasised that the mutation in *tmod4* is causative of the *tmod4^trg^* phenotype. Western blot analysis together with the reduced level of *tmod4* transcript, caused by nonsense-medicated decay, suggests that *tmod4^trg^* carries a null allele of *tmod4*. Importantly, *tmod4* has not been associated with human muscle disorders to date.

In mammals, four tropomodulin genes have been discovered, but in zebrafish only three are present: *tmod1*, *tmod3* and *tmod4*. Although zebrafish *tmod3* has been designated *tmod2* after the neuronal isoform, analysis of its amino acid sequence predicts it as an orthologue of *tmod3* (Yamashiro et al., 2012). Interestingly, sarcomere structure in *Tmod1* knockout mice remains preserved as Tmod1 function is partly replaced by Tmod3 and Tmod4 (Gokhin et al., 2010). Also in the zebrafish *tmod4^trg^* mutant, organised sarcomeres were formed, which could point to partial replacement of Tmod4 function by other Tmod proteins. However, organised sarcomeres were rarely detected and, in addition to defective sarcomeres, areas of scattered filaments were frequently observed in *tmod4^trg^* mutants, suggesting that functional replacement of Tmod4 was severely limited. Therefore, it could be speculated that the partial overlap of Tmod function could account for some of the broad variation of symptom severity in human muscle disorders.

The *tmod4^trg^* mutant is different to previously reported zebrafish models for dystrophic diseases that feature muscle atrophy characterized by myofibre degeneration (Berger and Currie, 2012). Dystrophic zebrafish display a patchy reduction in birefringence as stochastic myofibre degeneration causes loss of whole myofibres together with their birefringent myofibrils (Berger et al., 2012). By contrast, the birefringence of *tmod4^trg^* mutants was uniformly reduced throughout the trunk musculature, indicating deficiencies in the birefringent myofibril directly, rather than loss of whole myofibres. Similarly, on H&E-stained sections, myofibres appeared disorganised without signs of degeneration, also pointing to a myopathy-like phenotype. Haematoxylin-stained nuclei of *tmod4^trg^* mutants appeared rounder in shape compared with those of siblings, an interesting observation that might suggest that flattening of nuclei requires the presence of normal amounts of myofibril. In addition, organised sarcomeres were rarely detected by using electron microscopy and in live *Tg*(*acta1:lifeact-GFP*) transgenic *tmod4^trg^* mutants. Accordingly, *tmod4^trg^* showed drastically lower active force generation with altered responses at short muscle length and impaired muscle relaxation. Muscle weakness and absence of dystrophic signs are also characteristics of individuals suffering from severe myopathy, indicating that the discovered zebrafish mutant *tmod4^trg^* might be used as a novel model for human myopathies.

Sarcomere organisation in *tmod4^trg^* mutants was often characterized by abundant electron-dense cytoplasmic rods that in shape, size, frequency and immune-reactivity matched the nemaline rods detected in muscle biopsies of human patients with NM. Similar to NM individuals, widened Z-disks were eminent in sarcomeres of *tmod4^trg^* mutants. As Tmods are known regulators of thin filament length and stability (Gokhin and Fowler, 2013; Littlefield et al., 2001), thin filaments in *tmod4^trg^* mutants were analysed. Transmission electron microscopy rarely documented organised sarcomeres, and filaments were often misoriented and dispersed throughout myofibres. Although thin filament length in organised myofibrils was comparable to that of siblings, their length in disorganised sarcomeres was variable. In addition, *tmod4^trg^* mutant sarcomeres often featured undefined Z-zones, supporting the notion of variable thin filament length. In addition, the ascending limb of the relationship between active force and length was altered in *tmod4^trg^* homozygotes. The thin filament length has been shown to affect the descending limb of the length-tension relationship (Granzier et al., 1991). However, the observed steeper shape in the ascending limb in *tmod4^trg^* can also reflect variations of myofilament length, causing altered mechanical interactions in the sarcomere (Gordon et al., 1966), possibly disorganised sarcomeres or alterations in length-dependent activation (Rüdel and Taylor, 1971). Nonetheless, these findings collectively indicate that the length of thin filaments plays an important role in sarcomere assembly and, furthermore, failure of thin filaments to assemble in sarcomeres might trigger the formation of cytoplasmic rods. Interestingly, mutations in nebulin (*NEB*), another crucial regulator of thin filament length, cause NM in individuals, and loss of nebulin function in zebrafish, as well as in mouse, leads to the formation of cytoplasmic rods that resemble nemaline rods (Bang et al., 2006; Ottenheijm et al., 2009; Telfer et al., 2012; Witt et al., 2006), which supports the notion that misregulation of thin filament length might trigger nemaline rod formation.

Interestingly, NM individuals with mild symptoms show a prevalence of nemaline rods in type I (slow) fibres that is lost in more severe cases (Malfatti et al., 2014). In zebrafish, slow fibres form a single superficial layer on the outside of the myotome and are thereby clearly separated from the fast muscle. In line with the fast muscle-specific expression of *tmod4*, cytoplasmic rods of *tmod4^trg^* homozygotes were restricted to the fast myofibres and devoid of the slow fibre type. As cases of mild NM show the opposite pattern, the identified *tmod4^trg^* mutant is more appropriate as a model of severe NM cases. This is supported by the drastic reduction in force generation that is evident in *tmod4^trg^* mutants.

In addition to the muscle weakness in *tmod4^trg^* mutants, cartilage malformations in the head were also noted, probably due to altered muscle-cartilage interplay. In line with this finding, severe facial muscle weakness in some individuals diagnosed with NM is also accompanied by jaw contractures and elongated faces (Malfatti et al., 2014).

In summary, the novel zebrafish mutant *träge* possesses a null mutation in *tmod4*. The phenotype of homozygous mutants features characteristics of nemaline myopathy, implicating *TMOD4* as a novel candidate gene for the human muscle disorder. Furthermore, *tmod4* loss-of-function analysis indicates that misregulation of thin filament length might play an important role in the formation of nemaline rods, making the *tmod4^trg^* mutant a valuable model to study nemaline myopathy.

## MATERIALS AND METHODS

### ENU screen

As described previously, 48 male adult zebrafish in TU background were treated with ENU (Berger et al., 2011). In a classic three generation screen, mutagenized F0 males were outcrossed to TU females to establish mutant carriers that were then outcrossed to generate F2 families. Offspring of at least ten F2-incrosses were screened under polarised light for a reduction in birefringence at 3 dpf. The identified mutant *träge* was outcrossed over five generations to clean the mutant line from background mutations before phenotypic analysis. All animal experiments were approved by Monash Animal Research Platform (MARP/2012/167).

### Quantification of birefringence

Birefringence of the musculature was quantified as described previously (Berger et al., 2012). In short, unbiased pictures of anaesthetised larvae were taken using the automated setup of the Abrio LS2.2 polarizing microscope (Cri). Subsequently, images were subject to densitometry analysis using the Fiji software by measuring the average grey values of the pixels of the first 20 somites. The obtained values were normalised against control siblings that were set to 100%. For statistical analysis, six siblings and six mutants or morphants from three independent clutches were analysed for their muscle birefringence. Data are represented as means±s.e.m. and statistical significance was determined by using Student’s *t*-test.

### Contractile function of muscle

Per genotype, six larvae were individually mounted at slack length between a force transducer and a puller, as described previously (Li et al., 2013). Whole larval preparations (including all trunk muscles) were then stimulated to give single twitches through electrical pulses of 0.5-ms duration (supramaximal voltage) and 2-min intervals. Between contractions, the length was stepwise increased from the slack length to a length above that giving maximal for active force. At each length, active contraction was recorded. One contraction was included at a length above optimal for active force (L_opt_), to ensure that the L_opt_ for active force was identified. The descending limb of the length tension relationship was not fully examined, because the preparations tended to break at lengths above L_opt_. For each larval preparation, the optimal length (L_opt_) for peak active force and the time to reach half-maximal (t_1/2_) contraction and relaxation were determined. All experiments were performed using physiological buffered solution at 22°C.

### Mapping of *tmod4^trg^* mutants

*Träge* mutants, outcrossed over five generations in the TU background, were outcrossed to WIK wild types to establish the F1 mapping cross. Offspring from one mapping pair was phenotyped at 3 dpf, and 25 siblings and 25 mutants were pooled. Genomic DNA of each pool was extracted and sequenced using an Illumina HiSeq 100-bp paired-end sequencer (Illumina). Generated sequencing reads were used for linkage analysis with the software SNPtrack, and results were visualized in IGV as described previously (Leshchiner et al., 2012; Robinson et al., 2011). Genes in the linked homozygosity interval were amplified by using PCR and re-sequenced to verify a SNP in exon 5 of *tmod4* that resulted in a nonsense mutation.

### Genotyping

Two independent PCR-based assays were designed to genotype *tmod4^trg^* mutants for the identified SNP in *tmod4*. Whole embryos or clipped fins were degraded in 100 μl of 50 mM NaOH at 95°C for 15 min and the pH was adjusted afterwards by the addition of 25 μl of 1 M Tris pH 8.0. Kompetitive Allele Specific PCR (KASP) was performed using three competitive allele-specific primers that were fluorescently labelled: tmod4-wt (5′-GCTCATGAGTGTGTACATTCCCT-3′) anneals to the wild-type *tmod4* allele, tmod4-trg (5′-TGCTCATGAGTGTGTACATTCCCA-3′) targets the mutant sequence and tmod4-rev (5′-TGGAATTATGTTTGTCTCTTCACAGCCAT-3′) was used as the common primer. Subsequent endpoint fluorescent reading was achieved using the Synergy Mx instrument to identify the genotype (BioTek). A second independent PCR assay with the primer pair tmod4-F (5′-GGCTGTTACAGTGTTAGTTAAGTGGTT-3′) and tmod4-XbaI-R (5′-GTTGCTCATGAGTGTGTACATTCtC-3′) was undertaken. Digestion of the resulting 111-bp amplicon with the restriction enzyme *Xba*I cleaves only the amplicon of the mutant allele into 83-bp and 28-bp fragments, as revealed by using DNA electrophoresis.

### Morpholino injections

A morpholino antisense oligonucleotide targeting the translation start codon of *tmod4* was ordered from Gene Tools LLC with the sequence 5′-TCTGGGATCACTCTTAGACATACTT-3′. As described previously, 1.4 nl of tmod4_ATG(−4+21) was injected at a concentration of 200 μM into the yolk of one-cell-stage wild-type embryos (Berger et al., 2011). Siblings were injected with injection buffer as controls.

### Transgenic zebrafish lines

Expression plasmids (pActa1-mCherryCAAX-pA and pActa1-lifeact-GFP-pA) were assembled using the Gateway cloning system (Invitrogen) as described previously (Jacoby et al., 2009). pME-lifeact-GFP (accession no. JN717248) was constructed by using PCR subcloning, incorporating the LifeAct tag into the 5′ oligonucleotide (Riedl et al., 2008). Transgenic lines were produced as previously reported (Berger and Currie, 2013), and the strains *Tg*(*acta1:lifeact-GFP*) and *Tg*(*acta1:mCherryCAAX*) were outcrossed until Mendelian ratios were achieved. Genotyping was accomplished by using fluorescence analysis. The generation of transgenic lines was approved by the Institutional Biosafety Committee of Monash University (PC2-N74/08).

### Histology, immunohistochemistry, western blotting and *in situ* hybridization

According to standard methods, 10-μm cryofrozen sections of 3-dpf-old larvae were stained with H&E and Gomori trichrome or subjected to immunohistochemistry analyses (Berger et al., 2010). Primary antibodies against dystrophin (1:20, Mandra1, DSHB), TMOD4 (1:200, 11753-1-AP, Proteintech) or actinin (1:1000, A7811, Sigma) were used; AlexaFluor-568-conjugated phalloidin was obtained from Life Technologies (1:1000, A12380). Fluorescence images were recorded using a Zeiss LSM 510 Meta fluorescence confocal microscope (Zeiss, Germany). For electron microscopy, 3-dpf-old larvae were fixed in 2.5% glutaraldehyde in 0.1 M sodium cacodylate overnight at 4°C. Electron micrographs of ultrathin sections were taken on a Hitachi H7500 transmission electron microscope (Hitachi, Japan). *In situ* hybridization was undertaken on whole mounts using a *tmod4* digoxigenin-labelled antisense RNA probe that was synthesized using the DIG RNA labelling kit (Roche, Germany) and the plasmid IRBOp991E0136D (Source BioScience, UK) containing the *tmod4* cDNA.

## Supplementary Material

Supplementary Material
