# Supplementary material for: Loss of Tropomodulin4 in the zebrafish mutant träge causes cytoplasmic rod formation and muscle weakness reminiscent of nemaline myopathy
Source: Dis Model Mech. 2014 Oct 2;7(12):1407–15. doi: 10.1242/dmm.017376 (PMC4257009; doi:10.1242/dmm.017376)
Supplement: Supplementary Material [file supp_7_12_1407__index.html]

Loss of Tropomodulin4 in the zebrafish mutant träge causes cytoplasmic rod formation and muscle weakness reminiscent of nemaline myopathy — Supplementary Material 

# Loss of Tropomodulin4 in the zebrafish mutant *träge* causes cytoplasmic rod formation and muscle weakness reminiscent of nemaline myopathy

## DMM017376 Supplementary Material

**Files in this Data Supplement:**

- **Supplementary Material**
