# Supplementary material for: Loss of Tropomodulin4 in the zebrafish mutant träge causes cytoplasmic rod formation and muscle weakness reminiscent of nemaline myopathy
Source: Dis Model Mech. 2014 Oct 2;7(12):1407–15. doi: 10.1242/dmm.017376 (PMC4257009; doi:10.1242/dmm.017376)
Supplement: Supplementary Material [file supp_7.12.1407_DMM017376.pdf]

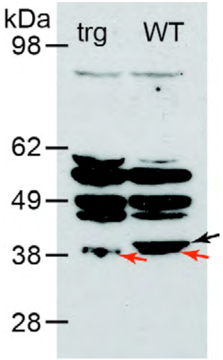

Figure S1: Western blot analysis using polyclonal antibody against human TMOD4. At 39kDa, the theoretical molecular weight of zebrafish Tmod4, a band appears in wildtype (WT) that is absent in the *tmod4*^{trg} mutant (trg), marked by black arrow. Red arrows mark bands of slightly lower molecular weight in both genotypes, likely representing Tmod1 or Tmod3 that are similar to Tmod4 in molecular weight and sequence. Compared to human TMOD4, zebrafish Tmod4 is 74%, Tmod1 is 56%, and Tmod3 is 57% identical.

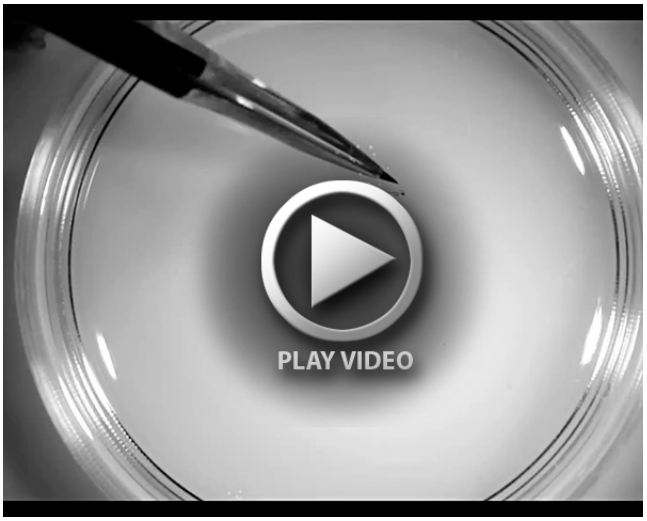

Supplemental movie S1: Startle response is triggered in siblings by a mechanical stimulus.

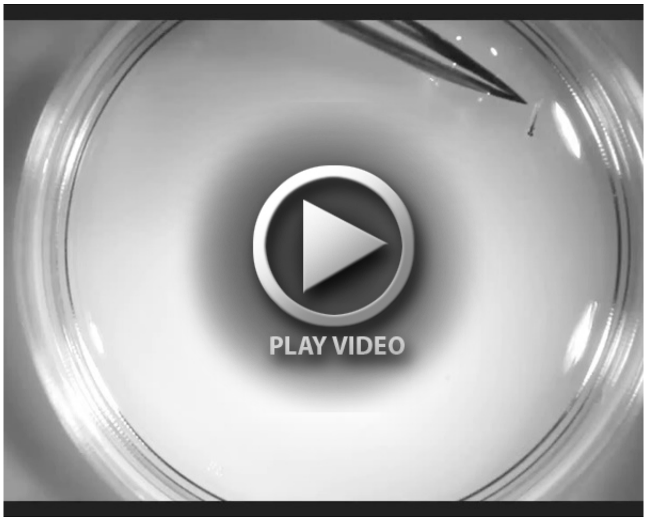

Supplemental movie S2: Startle response is triggered in *träge* by a mechanical stimulus. While the mutant reacts to the stimulus, the swimming behaviour is impaired with compromised forward thrust.
